# Clinical pharmacists’ intervention on pain management in cancer patients (PharmaCAP trial): study protocol for a randomized controlled trial

**DOI:** 10.1186/s40545-022-00505-0

**Published:** 2023-01-24

**Authors:** Sunil Shrestha, Ali Qais Blebil, Siew Li Teoh, Simit Sapkota, Bhuvan KC, Vibhu Paudyal, Siew Hua Gan

**Affiliations:** 1 School of Pharmacy, Monash University Malaysia, 47500 Subang Jaya, Selangor Malaysia; 2 Department of Clinical Oncology, Kathmandu Cancer Center, Tathali, Bhaktapur, Bagmati Province, Nepal; 3 Department of Clinical Oncology, Civil Service Hospital, Minbhawan, Kathmandu, Bagmati Province, Nepal; 4 College of Public Health, Medical and Veterinary Sciences, James Cook University, Townsville, QLD Australia; 5 Faculty of Pharmacy and Pharmaceutical Sciences, Monash University Parkville Campus Parkville, Melbourne, VIC 3052 Australia; 6 School of Pharmacy, College of Medical and Dental Sciences, Sir Robert Aitken Institute for Medical Research, University of Birmingham Edgbaston, Birmingham, B15 2TT UK

**Keywords:** Cancer pain, Cancer patients, Clinical pharmacist, Feasibility study, Pilot trial, Randomized controlled trial

## Abstract

**Introduction:**

Evidence-based services to support cancer patients with pain via clinical pharmacy services are currently lacking. Therefore, there is a need to undertake a randomized controlled trial (RCT) to explore the effectiveness of clinical pharmacists (CPs)’ input into the multidisciplinary team (MDT) in providing better therapeutic outcomes for cancer pain management.

**Objectives:**

The main aim of this pilot RCT is to determine the feasibility and preliminary efficacy of integrating CPs into the MDT for cancer pain management on the clinical outcomes of cancer patients experiencing pain.

**Methods:**

This study protocol outlines two-armed multicenter pilot RCT. Cancer patients suffering from pain will be randomly allocated to receive either clinical pharmacy services, i.e., PharmaCAP trial intervention from the CP, or the usual standard care (i.e., control group). Patients will be recruited consecutively from two hospitals in Kathmandu valley, Nepal. The outcomes will be assessed at baseline (pre-intervention) and 4 weeks post-intervention. The primary feasibility outcomes will include eligibility rate, recruitment rate, willingness to participate, acceptability of screening procedures and random allocation, possible contamination between the groups, intervention fidelity and compliance, treatment satisfaction, and patient understanding of the provided interventions. Subsequently, the primary clinical outcome, i.e., pain intensity of cancer patients, will be assessed. The secondary clinical outcomes will include health-related quality of life (HRQoL), anxiety, depression, adverse drug reactions, and patient medication compliance following the integration of CP into the healthcare team.

**Discussion:**

The feasibility and potential for integrating CP involvement in MDT to improve clinical outcomes of cancer patients with pain will be evaluated through the PharmaCAP trial.

*Trial registration*: ClinicalTrials.gov NCT05021393. Registered on 25th August 2022.

**Supplementary Information:**

The online version contains supplementary material available at 10.1186/s40545-022-00505-0.

## Introduction

According to the International Association for the Study of Pain (ISAP), pain is defined as *“an unpleasant feeling and emotional experience related to actual or potential tissue damage”* [1]. Pain remains one of the most common symptoms of cancer patients affecting approximately 66% of cancer patients [2]. Pain can affect the patient’s health-related quality of life (HRQoL) [3–5], and increase the use of health services, length of hospital stays, and overall healthcare costs [6]. It also has a negative impact on physical, psychological, and social activities of patients [3, 7, 8].

Prior literature suggests the important potential involvement of pharmacists as multidisciplinary team (MDT) members in managing cancer pain in terms of improving HRQoL, reducing medication non-compliance, and adverse drug reactions (ADRs) [9–11]. In preparation for this trial, the investigators of this study conducted a systematic review and meta-analysis [12]. The pharmacological and non-pharmacological interventions provided by pharmacists to help cancer patients experiencing pain were highlighted by the systematic review and meta-analysis of 12,684 cancer patients from 64 studies [12]. The most common interventions offered by pharmacists included medication review, patient education and counseling, ADRs detection and management, recommendations to the physicians (e.g. change in dose/regiment), and cancer pain assessment (either exclusively delivered by pharmacists or in collaboration with other healthcare professionals). This systematic review and meta-analysis also showed that pharmacists’ intervention significantly reduced pain intensity [pharmacist involvement group (IG) and control group (CG) [standardized mean difference of 0.35 (95% confidence intervals: − 0.55, − 0.16)] [12]. However, limited randomized controlled trials (RCTs) have assessed the impact of CP on pain intensity, HrQOL, ADRs, medication adherence and clinical outcomes prospectively considering the characteristics of cancer patients with pain.

## Rationale for study

Globally, cancer is the second leading cause of death and a significant contributor to the disease burden [13–15]. Various studies and research projects show that the global cancer burden will continue to grow for at least the next two decades [14, 16–18]. In a systematic analysis for the Global Burden of Disease Study 2019, there were an estimated 23.6 million new cancer cases and 10.0 million cancer deaths globally, with an estimated 250 million (235–264 million) Disability-Adjusted Life-Years (DALYs) due to cancer [16]. Cancer is a significant public health issue in Nepal, accounting for 10% of all fatalities [19, 20]. To date, both the incidence and mortality of cancer are increasing in Nepal, negatively impacting DALYs. The top five primary cancers and their causes of death in Nepal are lung, cervix, stomach, breast, and head and neck (lip, mouth, pharynx, larynx) cancers [21].

Working on the assumption that clinical pharmacist (CP)’s intervention offers positive outcomes in patients, a definitive RCT is needed to investigate the effects of the CP intervention among cancer patients experiencing pain. Currently, there is a lack of studies undertaken in low and middle-income countries (LMICs) to merit progression into the main RCT [12]. Pharmacists and CP in LMICs, such as Nepal, have limited roles and services in hospitals, and are not integrated adequately into the health systems. From a practice point of view, clinical pharmacy practice remains in its infancy in Nepal [22, 23], including their roles in cancer hospitals and managing cancer pain in Nepal. Therefore, a feasibility pilot study is needed to test trial procedures before undertaking a definitive RCT.

To date, hospital pharmacists in Nepal mainly focus on dispensing as well as counseling of dispensed medications, stock and inventory management. Some essential clinical pharmacy services provided by pharmacists and CPs in hospitals include medication review, clinical ward rounds, patient counseling, drug information, and pharmacovigilance activities [22, 23]. Hence, the pilot multi-center RCT attempts to introduce and emphasize the role of CP in cancer pain management as a clinical “Pharmacist intervention of CAncer Pain management” (PharmaCAP trial). PharmaCAP trial seeks to deliver multifaceted clinical pharmacy services to improve cancer pain outcomes and assess whether the CP can be integrated into collaborative care in oncology settings. In other words, the pilot RCT will address the potential for a CP intervention and associated study procedures in an innovative oncology settings for a definitive RCT in the future.

This study will involve a pilot RCT of CP intervention to determine whether their integration in an MDT is feasible and whether such integration has the potential to improve pain management outcomes for cancer patients compared to the usual standard care with no CP integration. Overall, the pilot RCT aims to determine the feasibility and efficacy of a CP intervention to reduce pain intensity, improve HRQoL, reduce ADRs and improve medication compliance among cancer patients. The CP’s intervention includes pharmacological (medication review, ADRs detection and management) and non-pharmacological intervention (patient education, counseling, pain assessment). The pilot multi-center RCT will also help compute a definitive trial sample size feasible for the PharmaCAP trial to deliver whether cancer patients will find the intervention acceptable. In addition, quantitative and qualitative feasibility assessments will be undertaken to help researchers proceed with the RCT to assess the effect of this intervention.

## Objectives and hypothesis

The primary objective of the pilot RCT is to evaluate the impact of the intervention (PharmaCAP trial) on the clinical outcomes of patients suffering from cancer pain or related pain.

The secondary objectives of the pilot RCT are to:

1. determine the number of cancer patients willing to participate in the study and receive the CP intervention.
2. estimate recruitment and attrition rates, as well as the 4-week follow-up rates.
3. evaluate the acceptability of CP for cancer pain management, elements of the intervention that were considered beneficial (or not), and appropriateness of study procedures using semi-structured interviews with participants, medical oncologists, and healthcare professionals.
4. investigate the acceptability of the intervention among the stakeholders by determining whether there are changes to medication regimens 4 weeks following the study.
5. explore the preliminary efficacy of the PharmaCAP trial to reduce pain intensity, enhance QoL, reduce ADRs, and improve medication compliance.
6. feasibility of collecting primary outcome data for the main trial

We hypothesize that cancer participants suffering from pain, randomized to the PharmaCAP trial intervention group (IG), will show a greater reduction in pain intensity at 4 weeks than those assigned to the control group. It is also hypothesized that the PharmaCAP trial intervention group will experience an enhanced HrQOL at 4 weeks compared to the control group (CG).

## Methods

### Study design

**Fig. 1:**
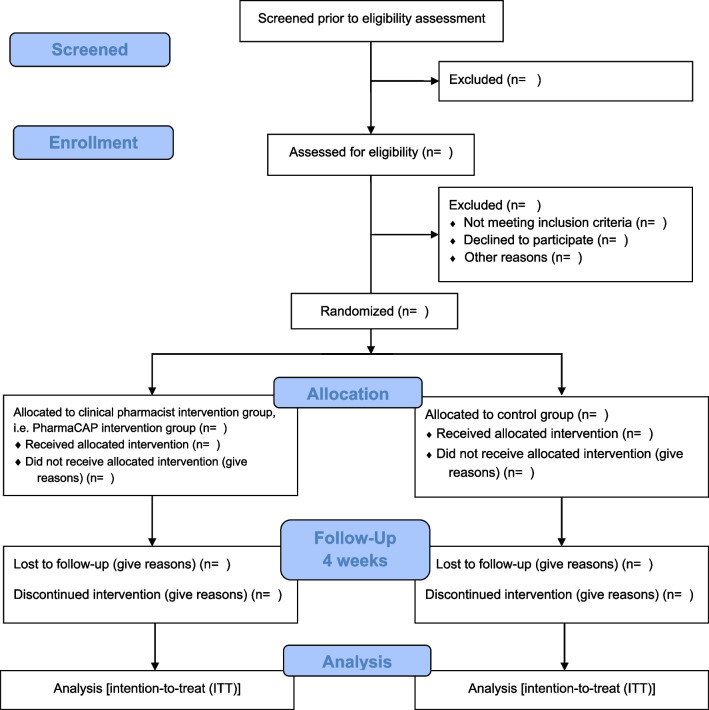
Consort flow diagram of the study

An open-label, two-arm, parallel-group pilot multi-center RCT with a 4-week follow-up period will be conducted to investigate the potential impact of CP integration into MDT. The two arms will include (1) an active arm where cancer patients suffering from pain will receive clinical pharmacy services from the CP (PharmaCAP trial) and (2) a control arm where cancer patients will receive the usual care (no intervention from a CP). Nevertheless, the research team members of this study are aware of the possibility of treatment contamination since both groups of cancer patients will attend the same hospital pharmacy to receive their medications. Although a cluster-randomized design will help resolve this issue, it is not applicable due to the complex intervention from CP, integration to MDT and the lack of a similar setup/settings in both sites. Instead, one CP will be recruited in the study to minimize the risk of potential contamination to provide care to the patients at each study site. Patients in the intervention group will receive multifaceted clinical pharmacy services from trained CPs, and those in the usual care will receive regular care from the hospital pharmacist as per the previous guide (Fig. 1). The study will be reported as per the reporting tool guideline, adapted for the pilot studies as proposed by Eldridge et al. [24], O’Cathain et al. [25] and the Standard Protocol Items: Recommendations for Interventional Trials (SPIRIT) reporting template (see Additional file 1 for SPIRIT 2013 checklist) [26].

The pilot trial is designed to address the specific objectives of the study design for a full trial to determine the preliminary effectiveness of the PharmaCAP trial. After that, data will be collected at two-time points (1) baseline (pre-intervention) and (2) following 4 weeks of intervention (post-intervention).

### Study setting and site

The study will be conducted at two hospitals of Kathmandu Valley, Nepal, i.e., (1) Kathmandu Cancer Center, Bhaktapur, Nepal, and (2) Civil Service Hospital, Kathmandu, Nepal. The former is an oncology-based hospital situated at Bhaktapur, while the latter is a tertiary care government hospital located in Kathmandu, Nepal. Three districts, Kathmandu, Bhaktapur, and Lalitpur, are in the Bagmati Province and have a combined population of almost 30 million people and a total area of 902.61 km^2^ (348.50 sq. mi). The capital city of Nepal is Kathmandu, and these three districts are in the country's central region. Due to adequate cancer treatment facilities in Nepal, Kathmandu valley has a higher patient flow. Therefore, the current study is only conducted in these study sites in Kathmandu valley. Eligible patient recruitment and data collection will be performed between May 2022 and November 2022.

### Participant and recruitment

Cancer patients who visit the study sites and seek pain management will be invited to participate in this study based on the clinical oncologists' recommendations for patients suffering from pain. A trained research team member will screen patients interested in participating in the study. Potentially eligible participants will be approached with written and verbal information provided by a trained research team member.

### Inclusion/exclusion criteria

Cancer patients aged 18 years and above will be included if they (1) have active cancer (any type), (2) have **s**elf-reported cancer pain up to a month before the enrollment into the study, (3) are receiving standard analgesic treatments or as one of the interventions could be to prescribe analgesics to those who are not currently taking, (4) are estimated to have more than 2 months of survival time, (5) can read and understand Nepalese or English language, (6) have access to either telephone or mobile phone and (7) can understand study information and sign written informed consents. Patients will be excluded if they (1) have moderate or severe cognitive impairment (as determined by the primary clinical oncologist), (2) have a severe vision or hearing impairment, (3) are involved in drug abuse, drug addiction, or alcohol dependence, (4) are unable to complete pain assessment; (5) are critically ill or patients on palliative care and those with opioid allergies that may restrict adherence to the trial procedures and (6) currently participate in any other investigational treatments or other study procedures that may influence their pain intensity.

Following adequate time to decide on study participation, a recruitment visit appointment will be organized, where written informed consent is obtained by the CP. Eligible cancer patients with pain will then be enrolled in the trial and be randomly assigned to either one of the two study groups. All participants in the IG will receive intervention from CP as a part of MDT, and those in the CG will receive the usual treatment. All participants will be assessed at baseline and 4 weeks following treatment. Details describing the schedule of the enrollment, interventions, and assessment are presented in Table 1 in the manner recommended by the Study Design, Population and Intervention (SPIRIT) checklist [27].

**Table 1:**
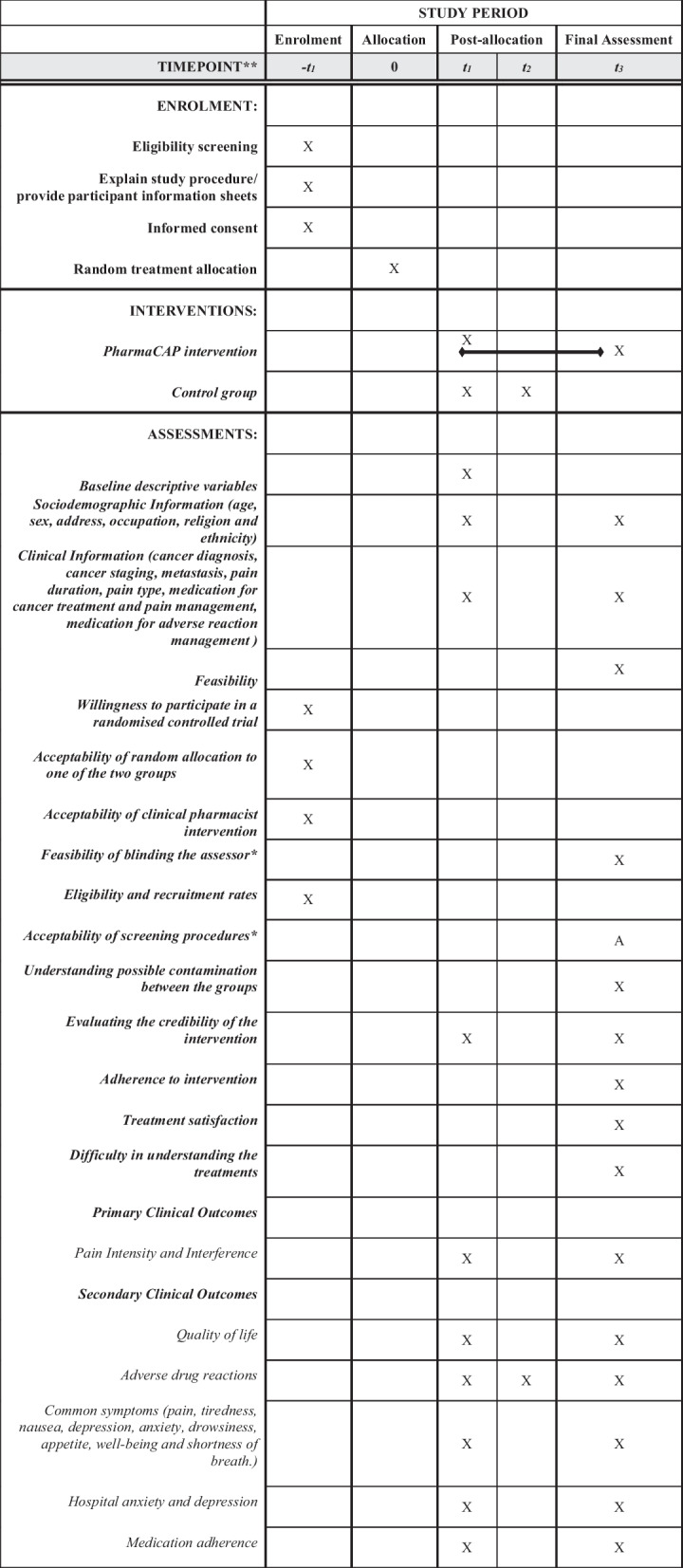
Schedule of enrollment, assessment and interventions

*Assessed by the clinical providing intervention; all other outcomes are assessed by the blinded outcome assessor. A Assessment at the end of every week on Fridays. *ADRs* adverse drug reactions

### Sample size

Sample size calculation is generally not mandatory for a pilot study [28]. However, the sample size was calculated by assuming an alpha value of 0.0500; (two-sided) power = 0.8000; alternative m = 1.7, and SD = 2.16585, and by considering the design of a similar published study [10]. The total sample size, including both groups, was calculated to be 26 patients. Allowing for a 30% attrition or drop-out rate, the minimum number of 34 cancer patients (17 participants/group) will be enrolled.

### Sample recruitment procedures

Cancer patients will be recruited consecutively at both study sites. Eligible patients must sign the written informed consent forms before the study procedure. The research team member will support patients with limited literacy or who request such support. Researchers will verbally explain the study protocol and assist in filling out the questionnaires (when required). Patients will be identified based on their symptoms following recommendations by the treating clinical oncologist. Additionally, hospital medical records of potentially eligible patients will be cross-checked for verification by the clinical research team to ensure that the patients included were as in the inclusion/exclusion criteria. Eligible patients will be referred to the researcher and invited for face-to-face interactions for participation in the study. During the first interaction, the purpose of the study will be explained to the interested patients, written informed consent will be obtained, and baseline assessments will be conducted.

### Randomization and blinding

A simple random sampling technique will be used from a list of eligible patients' random numbers through a computer-generated randomization sequence, using Microsoft Excel® (version 365). Randomization will be executed after the patients' consent and baseline assessment to avoid biases in the baseline findings. Randomization will be performed by requesting patients to handpick a sealed envelope from the basket indicating the allocation either to the (1) usual medical care or (2) CP intervention group with 1:1 randomization. Treatment assignments will be concealed in the envelopes prepared by a nurse (clinical team member) not directly involved in the study. The recruiting team will be involved in the subsequent intervention delivery.

Due to the nature of the study, it is impossible to blind the subjects to their respective treatment assignments. Therefore, there may be a high risk of social desirability bias as participants will not be blinded to their allocation of IG or CG. To minimize bias, the self-reporting instrument will be validated before implementing it for data collection [29]. However, treatment assignments will be concealed from the investigators (who will be different from the recruiting researcher) and the data analyst.

### Description and delivery of the trial intervention

All patients will be monitored for 4 weeks following the baseline assessment. Cancer patients (PharmaCAP trial intervention group) will have two face-to-face visits at baseline and during the follow-up assessment point (at week 4). Subsequent interventions will be conducted by the CP once a week by telephone over 4 weeks or face-to-face during the follow-up visits with the treating medical oncologists during hospitalization or emergency department visits (if any). A second assessment will be conducted at the end of week 4.

The CG will also undergo a similar baseline assessment, although they will receive the usual care (without CP intervention) during the remainder of the 4 weeks. In addition, the patient’s medical records will be accessed to obtain their socio-demographic, disease information, vital signs, medications for cancer treatment, medications for pain management and laboratory data. In both groups, patients will also be required to complete five questionnaires [comprising Brief Pain Inventory-Short Form (BPI-SF) [30–32], Edmonton Symptom Assessment System Revised (ESAS-r) [33, 34], Medication Adherence Report Scale (MARS‐5) [35], Hospital Anxiety and Depression Scale (HADS) Nepalese Version [36] and European Organisation for Research and Treatment of Cancer Quality of Life Questionnaire Core 30 (EORTC QLQ-C30)] [37] at two-time points (1) baseline visits and (2) study completion.

### Control group

Patients randomized to the control arm will receive the usual care from the hospital pharmacist during routine medication dispensing. Patients in this arm will continue to visit the hospital pharmacy to fill their prescriptions without further CP intervention. However, there will be no restriction on contacting the pharmacist for advice should they wish to. There will, however, be no other interference from the CP.

### Description of the intervention group (PharmaCAP trial)

In addition to usual care, cancer patients randomized to the PharmaCAP trial intervention group will receive multifaceted clinical pharmacy services and individualized pharmaceutical care from the CPs. The CPs will be integrated into the regular medical team in oncology settings, who will conduct interventions on the cancer patient’s treatment at other times for 12 hours/week for 6 months per practice preference. As usual, participants will continue to receive medical care from their oncologists, palliative care providers, primary care physicians, and other care teams. In addition to medical care, they will receive clinical pharmacy services and individualized pharmaceutical care from the CPs. Additionally, the CPs will conduct an initial face-to-face visit to complete a comprehensive medication review and to fill the baseline questionnaires at the study site immediately after the following randomization.

For the IG, medication review, patient education, counseling, recommendation and pain assessment will be performed by CPs. Patient education and counseling will include education regarding drugs used in pain management and their ADRs. Medication review includes assessing the appropriateness of each of the regular medications based on the diagnosis, laboratory findings, pain assessment, medication lists, consultation and discharge notes, procedures, and test results. Face-to-face assessment will be conducted on the patients at baseline and after an intervention (4 weeks). The CPs will assess the history of medication use for pain management, identify drug therapy-related problems (DTRPs), identify and manage ADRs (consists of detecting potential ADRs, providing and documenting appropriate follow-up until the ADR has resolved), and provide drug therapy interventions through written pharmacist notes to physicians/oncologists. These activities will be undertaken during the follow-up based on the medication chart review and the above pharmaceutical assessments. Patients will also receive general information about medication used for cancer pain management, potential adverse effects, and preventative strategies both in oral and written forms.

After the follow-up, the CPs will (1) educate patients on DTRPs identified before the visit, (2) reinforce the physician/oncologist's instructions, and (3) encourage medication compliance using written patient educational leaflets or verbal counselling. Telephone follow-ups will be conducted after the visit. Patients randomized to the CG will attend the medical follow-up and receive the usual care. All patients will be monitored for 4 weeks post-intervention. Data collection will be conducted at baseline and 4 weeks post-intervention. Patients will be encouraged to keep in touch with the CP through various communication tools, including short messages, mobile phone contact, Viber®, or WhatsApp®. They will also be encouraged to request a consultation with CP for any issues with their pain control or pain medication. Additional clinic visits/phone calls will be arranged based on the patient’s needs. During follow-ups, the study’s CP will clinically assess patients' understanding of their medications, review medications and home monitoring (pain), identify and resolve any new DTRPs, and provide additional education, if required. The CPs will also document patient visits and phone calls in the patient's medical record (Table 2).

**Table 2:** List of activities that clinical pharmacists will conduct in PharmaCAP intervention group

Activities that clinical pharmacists will carry out	Comment
Medication review	• Assessing the appropriateness of each of the regular medications used for cancer pain management based on symptoms, laboratory findings, medication lists, consultation, and discharge notes, procedures • Test impetrations and therapeutic recommendations • Medication reconciliation and identification for appropriate monitoring
Patient education and counselling	• Educate patients on drug therapy-related problems identified before the visit, reinforce clinical oncologists/radiation oncologists/physician's instructions • Providing instruction about why and how to take opioids, common adverse drug reactions due to opioids and how to deal with them • Education regarding drugs used in pain management and management of adverse drug reactions due to cancer pain management medications • Addressing concerns and general counselling on the condition of cancer patients • Providing further insight into an overall plan with patients and caretakers
Recommendation	• Clinical pharmacist will give verbal recommendations for dose modification, drug modification, etc., to the medical oncologist. When medical oncologists are unavailable at the site, then written recommendations will be given to the medical oncologist
Pain assessment	• Assessment of cancer patient’s pain by the clinical pharmacist
Drug information	• Evidence-based answers to queries from multidisciplinary teams relating to medications • Liaising with cancer patients and their caregivers concerning medication alerts and related queries
Adverse drug reaction	• Monitoring adverse drug reactions and informing the medical oncologist • Detection of adverse drug reactions
Medication adherence	• Education to improve medication adherence among cancer patients • Encourage medication adherence using written patient educational leaflets • While the discharge of cancer patients providing pharmaceutical care services to reduce the medication discrepancies before and after discharge, and improve patient medication adherence and knowledge related to pain, pain medication and their adverse drug reaction
Ward round	• Participation in inpatient ward round alone or with MDT

### Plan of contact for those who do not attend

Patients from both groups (IG and CG) who do not respond will be contacted via telephone up to three times. If the patient does not respond, they will be deemed lost to follow-up and will not be included in the analysis.

### Outcomes

#### Primary outcomes

The primary outcome of this pilot study is the feasibility of the 4-week CP intervention for cancer patients suffering from pain. The primary outcomes (feasibility) will be assessed throughout the study duration. Feasibility measure includes (1) recruitment and completion rates (number of patients' referred, eligible, enrolled, withdrawals, trial recruitment rate, and trial completion rate); (2) treatment adherence (the number of completed sessions delivered by the CP and the missed sessions) while the recruitment and completion rates will be assessed during the entire trial process. In addition, patient safety and treatment adherence will be assessed during interventions. Face-to-face assessment will be administered at baseline (week 0) and after 4 weeks of intervention (at week 4).

#### Primary clinical outcomes and other clinical outcomes measures

The primary clinical outcome is the difference in pain intensity at 4 weeks compared to the baseline. The Brief Pain Inventory (Short form) [BPI-SF] questionnaire will assess pain intensity and interference among cancer patients [30–32]. Since there is no Nepalese version available, a translation, cross-cultural adaptation and validation will be conducted before starting this trial. Permission has been taken, and an agreement with the originator was signed to ensure the lawful use of the questionnaire.

#### Secondary clinical outcomes and outcome measures

The secondary clinical outcomes and outcomes measures are as follows:

- Health-related quality of life [European Organisation for Research and Treatment of Cancer Quality of Life Questionnaire Core 30 (EORTC QLQ-C30)] [37] will be used. Permission was taken for using the EORTC QLQ-C30 Nepalese version, and an agreement with the European Organisation for Research and Treatment of Cancer (EORTC) was signed to ensure the lawful use of the questionnaire.
- Nine common symptoms [Edmonton Symptom Assessment System Revised (ESAS-r)] [33, 34] will be assessed. Permission was taken for translation, cross-cultural adaptation, validation and its use (ESAS-r Nepalese Version), and an agreement with the originator was signed to ensure lawful use of the questionnaire.
- Anxiety and depression [Hospital Anxiety and Depression Scale (HADS) Nepalese Version] [36] will be assessed. HADS has been demonstrated to be reliable and valid as a screening tool in psychiatry. The Nepalese version, which is readily available, has satisfactory psychometric properties. Permission was taken from the Nepalese translator, who permitted to use the questionnaire.
- Medication adherence [Medication Adherence Report Scale (MARS‐5)] [35] will be assessed. As a measure for gauging compliance, the five-item MARS-5 has the potential for evaluating adherence, recognizing patients who report low compliance, and the particular types of non-compliance behaviors (e.g., forgetting or intentionally missing doses). Consent was taken for the translation, cross-cultural adaptation and its use, and an agreement with the originator was signed to ensure the lawful use of the questionnaire.
- ADRs related to cancer pain treatment and analgesics will be assessed according to the National Cancer Institute (NCI) Common Terminology Criteria for Adverse Events (CTCAE, version 4) [38]. Identification and management of ADRs consist of detecting potential ADRs. Documentation appropriate for follow-up will be provided until the ADR has been resolved.

### Additional measures

Additional questionnaires are developed by the research team after a literature review. These additional questionnaires developed will be administered to obtain information related to (1) socio-demographic information (age, sex, education level, employment status, monthly household income, religion, ethnicity, health insurance); (2) cancer status (cancer diagnosis, staging, history of cancer, comorbidity, Karnofsky Performance Scale (KPS), metastasis and its site, medicine used for cancer treatment, family history), and (3) pain history including duration of pain, pain flares, pain management index (PMI), aggravating and relieving factors, medicines used for cancer pain management as well as ADRs due to drugs used for cancer pain management. Other information, such as resources required to conduct the trial (e.g., cost) and the time expected to enroll the optimal number of participants, will also be documented [39]. Additionally, the total duration of providing intervention by CP in the PharmaCAP trial intervention will also be recorded. After the completion of the trial, participants in the PharmaCAP trial intervention group will be asked about their satisfaction with the services from the CP with “yes” or “no” questions such as “Are you satisfied with the clinical pharmacy services?” or “Did your CP help address drug-related queries?”.

### Fidelity assessment

Data collection and study implementation will be supervised via weekly meetings with the PI, research team, representatives from the study sites, research pharmacists, and project CPs to ensure that protocols of trial are reliably executed. Data collected in this study will be confidentially treated and securely stored at the School of Pharmacy, Monash University Malaysia. Only the study investigators at the School of Pharmacy, Monash University Malaysia, will access the final dataset.

### Data management

The research team will designate study participants with a unique code to facilitate data linkage during the follow-up. The research team members who are trained in data entry will enter all collected data. One of member from the research team will verify the data entry for all participants.

### Statistical analysis

An intention-to-treat (ITT) analysis will be conducted. Quantitative data will be imported into statistical package for social sciences (SPSS) version 28.0 (SPSS Inc., Chicago, IL, USA) for the data analysis. Baseline and demographic characteristics will be descriptively analyzed (i.e., the number of valid cases, mean, standard deviation, median, and interquartile range). Changes in the secondary outcome measures at follow-up will be assessed using paired t-tests (for continuous variables) and McNemar’s test (for categorical variables). A p-value < 0.05 will be considered statistically significant for all analyses.

### Confidentiality and data security

All required measures will be taken to preserve the confidentiality of the study participants and protect data. Access to the data will be under strict privacy and security throughout the study duration, publication, and at any public presentation. No identifiable data will be released. The data will be stored using codes assigned by the PI and supervisor and be kept on password-protected computers. The study database will only be accessible to those people whom the lead researcher of the study has given permission.

### Plan for supervision and monitoring

SS_a_ and SS_b_ will always supervise the study in the study sites to ensure that the study protocol is followed to maximize adherence, solve problems, and consider how to respond flexibly to the needs of each case. In continuing group supervision, research team meet fortnightly in a group format under the direction of AQB, SLT, BKC, VP and SHG. This includes exchanging case studies, discussion of problems, its solution and instructive insights with other researchers engaged in the related subjects. The entire team will handle low participation rate-related issues that arise throughout the trial. All the ethical principles provided by the Declaration of Helsinki will be followed by all the research members throughout the study. The investigators will not violate any rules and ethical codes of the Nepal Health Research Council (NHRC) and Monash University Human Research Ethics Committee (MUHREC). The investigators of this study will regulate the NHRC and MUHREC ethical principles among all research team members and research assistants involved in the study.

### Ethics and dissemination

Cancer patients suffering from pain who meet the inclusion criteria will be provided oral and written information regarding the trial objective. Following informed written consent, patients who do not wish to participate are free to decline or withdraw from the study at any time. Confidentiality of cancer patients’ responses and data will be assured. The study has been approved by the Ethical Review Board NHRC in Nepal (Ref No 768; Protocol Registration No. 497/2021) and MUHREC (Project ID: 30907). Ethical approval from institutional review committee and hospital management are also taken. The protocol trial is registered in the Clinical Trials Registry (ClinicalTrials.gov Identifier: NCT05021393).

Moreover, if cancer patients feel uncomfortable with the study trial, they are allowed to withdraw at any time. No agreements or other regulations will limit access to the data collected. The study will be reported according to the guidelines of the Consolidated Standards of Reporting Trials [24].

### Study status

This PharmaCAP trial is being undertaken between May to November 2022. Data collection will proceed as initially planned. Additionally, the CPs and research team will take necessary precautions when entering practices to collect the data and for patient intervention, including wearing personal protective equipment (PPE), considering an ongoing pandemic.

## Discussion

To the authors' knowledge, this is the first pilot multicenter RCT that is going to be conduct in Nepal that evaluates an integration of CP in MDT for managing cancer patients suffering from pain. The study will inform the development of definitive RCT and the development of evidence related to the involvement of CPs as essential members of the MDT for pain management in cancer patients.

In addition to being the first study in LMICs, such as Nepal, to evaluate the feasibility and acceptability of a CP among the cancer population with pain in the country, this study will also provide preliminary insights into the impact of a CP on cancer pain-related outcomes. Moreover, integrating CP to MDT takes an inherently patient-centered approach to pain management. This study will aid in generating evidence regarding the contribution of CP in cancer pain management and incorporating them into collaborative cancer pain management practice in more extensive multi-site settings.

CPs can contribute to cancer pain management by delivering pharmaceutical care, reducing pain intensity, and improving HRQoL. However, CP’s involvement in cancer pain management is new and necessitates assessment before organizing a large-scale RCT. PharmaCAP trial intervention will be a new practice in the case of LMICs, and the study will determine whether future definitive trials can be conducted. Findings will be disseminated through conference presentations and peer-review open-access publications. If the pilot RCT is feasible, acceptable to the stakeholders, and demonstrates a positive impact and cost-effectiveness, a full, pragmatic RCT will be conducted.

This study is novel and innovative in low-resource settings because CP’s role in cancer pain management will contribute to the treatment outcome of cancer patients and help their integration in MDT in a rigorously designed pilot RCT. Establishing the role of a CP is essential for improving clinical and therapeutic outcomes of cancer patients suffering from pain who may benefit from skilled hospital CPs, doctors in LMICs, and CPs.

### Implications of trial

The successful execution of a future full-powered RCT will be based on the current intervention delivered by the CP. Therefore, CP can potentially benefit cancer patients suffering from pain. CP can work alongside oncologists and other healthcare professionals in hospitals to help improve outcomes for cancer pain. The results of this trial will be of relevance to policy makers on the importance of CP in oncology settings.

### Strengths of trial

The pilot multicenter RCT is the first to be conducted in LMIC, Nepal, involving CPs who deliver a combination of pharmacological and non-pharmacological interventions to cancer patients suffering from pain. This study will help demonstrate the value of integrating CPs in MDT in an oncology setting to reduce pain intensity. Overall, it can help improve clinical outcomes in cancer patients.

### Limitations and barriers to the trial

The duration for the 4 weeks of intervention may be too short for showing the full influence of the intervention on cancer- and drug-related outcomes. Nevertheless, as this trial is a pilot study, the findings of this study will provide preliminary results regarding the efficacy of integrating CP in the MDT being tested. Although the study sample size will be sufficient to determine the viability of running a full-scale RCT, the sample size is not large enough to formally compare the outcomes of the two treatments. It will provide an estimate of the variation on which to power a future, definitive trial. Another potential barrier to a future trial is the lack of CP in cancer hospitals in medical oncology units and cancer hospitals of Nepal, and the ability to recruit CP for future trials. Future trials can be expanded to other cancer hospitals and institutions nationwide.

## Conclusions

The PharmaCAP trial is one of the first pilot multicenter RCTs to be conducted in Nepal to evaluate the feasibility of integrating CP in MDT and their impact on the clinical outcomes of cancer patients suffering from pain. The study will inform the design, procedures, measures, interventions delivered by the CP, and data analyses for a larger, multicenter trial to generate further robust evidence in future RCTs.

## Supplementary Information

**Additional file 1.** Recommended items to address in a clinical trial protocol and related documents.

## Data Availability

The data are available upon request with the corresponding author.
